# Legionella Septic Cardiomyopathy Requiring Venoarterial Extracorporeal Membrane Oxygenation Followed by Presumed Complement-Mediated Thrombotic Microangiopathy: A Case Report

**DOI:** 10.3390/jcm15197417

**Published:** 2026-09-24

**Authors:** Hui Liu, Chengduo Zhang, Shengjia Peng, Dan Li, Ming Cui

**Affiliations:** 1 Department of Cardiology and Institute of Vascular Medicine, Peking University Third Hospital, Beijing 100191, China; liuhui75321@163.com (H.L.); zhangchengduobjmu@163.com (C.Z.); 2411110547@stu.pku.edu.cn (S.P.); 2 State Key Laboratory of Vascular Homeostasis and Remodeling, Peking University Third Hospital, Beijing 100191, China

**Keywords:** *Legionella pneumophila*, septic cardiomyopathy, venoarterial extracorporeal membrane oxygenation, complement-mediated thrombotic microangiopathy, case report

## Abstract

**Background:** *Legionella pneumophila* rarely causes profound myocardial dysfunction and malignant ventricular arrhythmias. Persistent thrombocytopenia and acute kidney injury during recovery may additionally raise concern for thrombotic microangiopathy (TMA), although distinguishing complement-mediated disease from secondary causes in critically ill patients is challenging. **Case Presentation:** A 64-year-old man presented after five days of fever with recurrent ventricular tachycardia/ventricular fibrillation, cardiac arrest, and refractory shock. Echocardiography demonstrated diffuse biventricular hypokinesia with a left ventricular ejection fraction (LVEF) of 18%, while urgent coronary angiography showed no acute coronary occlusion. Venoarterial extracorporeal membrane oxygenation (VA-ECMO), intra-aortic balloon counterpulsation, and organ support were initiated. Levofloxacin was administered from admission, before sputum sequencing identified *L. pneumophila*. VA-ECMO was removed after four days. Persistent anaemia and thrombocytopenia with progressive acute kidney injury despite clinical improvement prompted evaluation for thrombotic microangiopathy. ADAMTS13 activity was 92%, soluble C5b-9 was elevated to 648 ng/mL, and schistocytes were not identified. Presumed complement-mediated TMA was treated with four weekly doses of eculizumab, followed by haematological and renal improvement. Sepsis, extracorporeal support, transfusions, and gastrointestinal bleeding confounded diagnosis and treatment attribution. At three months, LVEF was 59%, platelet count 237 × 10^9^/L, and creatinine 97 µmol/L. **Conclusions:** VA-ECMO may bridge selected patients with severe septic myocardial dysfunction to recovery. Persistent cytopenias and kidney injury warrant structured TMA evaluation. In critically ill patients, complement-mediated TMA requires integrated assessment rather than reliance on a single complement biomarker or clinical response to complement inhibition.

## 1. Introduction

Legionnaires’ disease is a severe community-acquired pneumonia with recognised extrapulmonary manifestations involving the neurological, hepatic, renal, skeletal-muscle, and cardiovascular systems [1,2]. Cardiac involvement is uncommon but includes arrhythmias, myocarditis, and sepsis-associated myocardial dysfunction [3,4]. Septic cardiomyopathy is a heterogeneous syndrome without universally accepted diagnostic criteria; diffuse ventricular dysfunction, reversibility, and exclusion of an acute ischaemic culprit support the diagnosis, although these features are not pathognomonic [5,6,7,8]. Complement-mediated thrombotic microangiopathy (TMA) is a further diagnostic challenge because its manifestations overlap with secondary TMAs and no single routine test establishes the diagnosis [9].

We report a case of severe *Legionella pneumophila* infection with reversible myocardial dysfunction and subsequent suspected complement-mediated TMA. The case illustrates the use of temporary circulatory support in a selected cardiogenic septic shock phenotype and the difficulty of attributing persistent haematological and renal abnormalities in critical illness.

## 2. Detailed Case Description

On 15 December 2025, a 64-year-old man with persistent atrial fibrillation, previous left atrial appendage occlusion, prior coronary stent implantation, and chronic edoxaban therapy developed high fever (maximum, 40 °C), chills, headache, myalgia, and fatigue. Respiratory symptoms were initially absent. Empirical ertapenem did not produce clinical improvement, and chest computed tomography (CT) on 19 December showed left-predominant pneumonia. During the following night, recurrent vomiting was followed by sudden loss of consciousness. Cardiac monitoring documented recurrent ventricular tachycardia and ventricular fibrillation (VT/VF), requiring repeated electrical defibrillation before sustained return of spontaneous circulation. Shock persisted despite norepinephrine 0.6 µg/kg/min and metaraminol 10 µg/kg/min.

On transfer to our emergency department on 20 December 2025, the patient was in atrial fibrillation at a ventricular rate of 150 beats/min, with a blood pressure of 85/73 mmHg and a serum lactate level of 7.2 mmol/L. Initial laboratory testing showed a haemoglobin level of 138 g/L, platelet count of 137 × 10^9^/L, serum creatinine of 186 µmol/L, procalcitonin of 33 ng/mL, troponin I of 1.4 ng/mL, and N-terminal pro-B-type natriuretic peptide of 22,800 pg/mL. The clinical course and major diagnostic and therapeutic events are summarised in Figure 1.

Transthoracic echocardiography demonstrated diffuse biventricular hypokinesia with a left ventricular ejection fraction (LVEF) of 18%. Emergency coronary angiography showed patent previously implanted stents and no acute coronary occlusion (Supplementary Figure S1). In the setting of severe infection, global ventricular dysfunction, and absence of an acute coronary culprit, septic cardiomyopathy was considered the most likely working diagnosis. Serial echocardiographic findings during the acute phase and early haemodynamic recovery are shown in Supplementary Figure S2. Representative serial chest CT images are provided in Supplementary Figure S3.

Peripheral venoarterial extracorporeal membrane oxygenation (VA-ECMO) was initiated on 20 December for refractory shock with profound myocardial dysfunction. During coronary angiography, poor aortic valve opening and delayed contrast clearance from the aortic root prompted intra-aortic balloon pump (IABP) placement for left ventricular unloading. Invasive mechanical ventilation and continuous renal replacement therapy (CRRT) were initiated concurrently. Levofloxacin was initiated on admission (20 December), providing empirical Legionella-active coverage, and piperacillin–sulbactam was also administered to broaden antibacterial coverage. Azithromycin was added on 21 December after multidisciplinary assessment raised suspicion of Legionella pneumonia. Sputum metagenomic and targeted next-generation sequencing identified *L. pneumophila* on 23 December. The existing regimen was continued given the favourable clinical response and microbiological findings. Haemodynamics and ventricular function improved rapidly, permitting VA-ECMO decannulation on 24 December, followed by discontinuation of IABP support and invasive ventilation on 26 December.

Despite haemodynamic and infectious recovery, thrombocytopenia, anaemia, and acute kidney injury persisted, prompting evaluation for TMA. ADAMTS13 activity was 92%, arguing strongly against thrombotic thrombocytopenic purpura due to severe ADAMTS13 deficiency. Soluble C5b-9 was elevated to 648 ng/mL, whereas C3 and C4 levels were within their reference ranges, and schistocytes were not identified on a peripheral blood smear. Longitudinal haematological and renal trajectories are shown in Figure 2.

The persistence of haematological abnormalities and severe kidney injury despite clinical improvement raised concern for complement-mediated TMA, clinically compatible with atypical haemolytic uraemic syndrome (aHUS). Both the diagnosis of TMA and its complement-mediated aetiology remained presumptive because sepsis-associated coagulopathy, recent VA-ECMO exposure, possible circuit-related haemolysis, transfusions, anticoagulation, and other critical-illness-related factors provided competing explanations. Anticoagulation was further complicated by thrombocytopenia, bleeding risk, and a suspected cardioembolic neurological event on 31 December. Eculizumab was administered at 900 mg weekly for four doses beginning on 1 January. No meningococcal vaccination was administered before eculizumab initiation. Systemic antibiotic treatment for severe pneumonia was ongoing at the time of the first dose and continued throughout the four-dose induction course. Peptic ulcer bleeding was confirmed endoscopically on 7 January, further confounding interpretation of the anaemia. Haematological and renal abnormalities subsequently improved (Figure 2).

By 22 January, platelet count and renal function had substantially improved. The patient was discharged on 23 January without further outpatient eculizumab. At the three-month follow-up on 27 March 2026, LVEF had recovered to 59%, with further improvement in haematological and renal parameters (haemoglobin 107 g/L, platelet count 237 × 10^9^/L, and serum creatinine 97 µmol/L).

## 3. Discussion

The initial absence of respiratory symptoms did not exclude Legionnaires’ disease: fever, headache, myalgia, and gastrointestinal symptoms may precede prominent respiratory manifestations [1,2]. The cardiac manifestations of Legionnaires’ disease range from arrhythmias and myocarditis to sepsis-associated myocardial dysfunction [3,4]. In this patient, profound global biventricular hypokinesia with an LVEF of 18%, the absence of an acute coronary culprit, and subsequent functional recovery were most consistent with septic cardiomyopathy [5,6,7,8]. Recurrent VT/VF reflected the severity of cardiac involvement. Nevertheless, Legionella-associated myocarditis could not be excluded because neither cardiac magnetic resonance imaging nor endomyocardial biopsy was performed. Septic cardiomyopathy was therefore considered the most likely clinical diagnosis, although the underlying mechanism of myocardial injury was not pathologically established.

The potential role of VA-ECMO in septic shock depends strongly on haemodynamic phenotype. Observational evidence suggests that patients with severe sepsis-induced myocardial dysfunction and a substantial cardiogenic component may derive greater benefit than those with predominantly vasoplegic shock [10,11]. In this case, profound biventricular dysfunction, recurrent malignant ventricular arrhythmias, refractory shock, and hyperlactataemia indicated a major cardiogenic component. Rapid haemodynamic and ventricular recovery permitted VA-ECMO removal after four days, consistent with its use as a temporary bridge to recovery in potentially reversible myocardial dysfunction. Poor aortic-valve opening prompted IABP insertion for left ventricular unloading, an important management consideration during peripheral VA-ECMO [12]. These findings support careful phenotype-based selection rather than routine use of VA-ECMO in undifferentiated septic shock. Briceño et al. reported myocardial recovery after temporary TandemHeart support in Legionella-associated myocarditis [4]. Both cases illustrate the feasibility of temporary mechanical circulatory support as a bridge to recovery; the present case involved peripheral VA-ECMO with IABP unloading in a septic-shock phenotype, although myocarditis could not be excluded. These reports do not establish comparative efficacy between support devices.

Persistent thrombocytopenia, anaemia, and kidney injury despite haemodynamic and infectious recovery raised concern for TMA, although determining its aetiology was challenging after critical illness and extracorporeal support. Evaluation requires integration of organ injury, thrombocytopenia, and evidence of microangiopathic haemolysis with assessment of secondary causes [13,14]. Complement-mediated TMA/aHUS remains a clinical diagnosis of exclusion; no single routinely available biomarker is sufficiently specific to establish it [9]. Preserved ADAMTS13 activity strongly argued against thrombotic thrombocytopenic purpura (TTP) associated with severe ADAMTS13 deficiency, but did not establish aHUS [15]. The absence of schistocytes reduced the evidence for overt microangiopathic haemolysis but did not, in isolation, exclude TMA [16,17]. Elevated soluble C5b-9 demonstrated terminal complement activation but did not distinguish complement-mediated TMA from complement activation associated with severe sepsis or extracorporeal circulation [18,19]. Normal C3 and C4 concentrations likewise neither excluded nor confirmed complement-mediated disease [20].

Accordingly, complement-mediated TMA, clinically compatible with aHUS, remained a working diagnosis rather than a definitively established aetiology. The persistence of renal and haematological abnormalities despite improvement in sepsis, together with evidence of terminal complement activation, supported consideration of C5 inhibition [20,21]. However, sepsis-associated coagulopathy, recent VA-ECMO exposure, possible circuit-related haemolysis, transfusions, anticoagulation, and gastrointestinal bleeding provided important competing or contributing explanations. Renal histology and definitive complement genetic evaluation were unavailable, further limiting aetiological confirmation. Although terminal complement inhibition is effective in established aHUS [21,22], haematological and renal improvement after eculizumab in this patient cannot establish either treatment efficacy or diagnostic certainty. Concurrently, infection control, withdrawal of extracorporeal support, renal replacement therapy, transfusion, and supportive care could each have contributed to recovery.

This case highlights two practical considerations. First, temporary mechanical circulatory support may provide a bridge to recovery in carefully selected patients with septic shock and profound, potentially reversible myocardial dysfunction. Second, persistent haematological and renal abnormalities that do not improve in parallel with infection and haemodynamics should prompt a structured evaluation for TMA rather than automatic attribution to sepsis or extracorporeal support. In critically ill patients with multiple competing causes of cytopenia, haemolysis, and kidney injury, supportive findings should be interpreted according to their specificity and within the overall clinical context. The absence of cardiac magnetic resonance imaging or myocardial biopsy limited the cardiac differential diagnosis, while the lack of renal histology and definitive complement genetic evaluation constrained assessment of TMA. Follow-up to three months cannot establish the long-term risk of relapse after stopping complement inhibition. The absence of pre-treatment meningococcal vaccination was a limitation of infection-risk mitigation; concomitant antibiotics for pneumonia do not establish that recommended preventive measures were fulfilled [23].

## 4. Conclusions

VA-ECMO may serve as a bridge to recovery in selected patients with septic shock and profound, potentially reversible myocardial dysfunction. Persistent haematological and renal abnormalities despite improvement in infection and haemodynamics should prompt structured evaluation for TMA. In critically ill patients with multiple competing aetiologies, complement-mediated TMA should be considered within an integrated diagnostic framework rather than inferred from a single complement biomarker or clinical response to complement inhibition. Prospective studies should refine selection for temporary circulatory support in septic myocardial dysfunction and evaluate integrated clinical, haematological, renal, and complement assessments for distinguishing secondary TMA from complement-mediated disease.

## Figures and Tables

**Figure 1:**
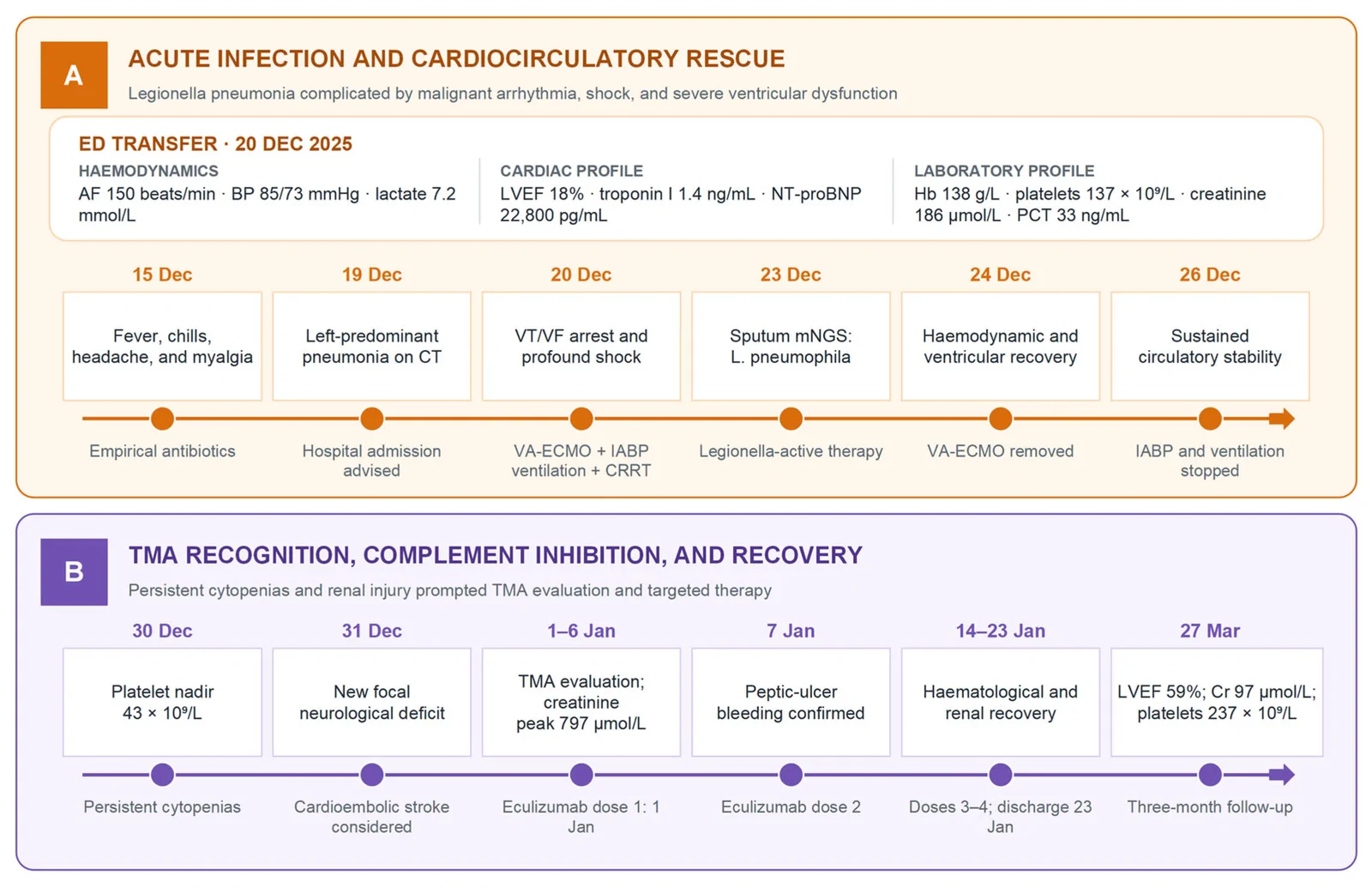
Timeline of the major clinical events, diagnostic findings, and therapeutic interventions. (**A**) Acute infection and cardiocirculatory support. (**B**) Evaluation for presumed complement-mediated thrombotic microangiopathy, eculizumab treatment, and recovery. Dates are shown explicitly; intervals are not drawn to scale. Legionella-active antimicrobial therapy began on 20 December 2025; the 23 December marker denotes microbiological confirmation and continuation of the existing regimen. Abbreviations: AF, atrial fibrillation; BP, blood pressure; Cr, creatinine; CRRT, continuous renal replacement therapy; CT, computed tomography; ED, emergency department; Hb, haemoglobin; IABP, intra-aortic balloon pump; LVEF, left ventricular ejection fraction; mNGS, metagenomic next-generation sequencing; NT-proBNP, N-terminal pro-B-type natriuretic peptide; PCT, procalcitonin; TMA, thrombotic microangiopathy; VA-ECMO, venoarterial extracorporeal membrane oxygenation; VT/VF, ventricular tachycardia/ventricular fibrillation. *L. pneumophila, Legionella pneumophila*.

**Figure 2:**
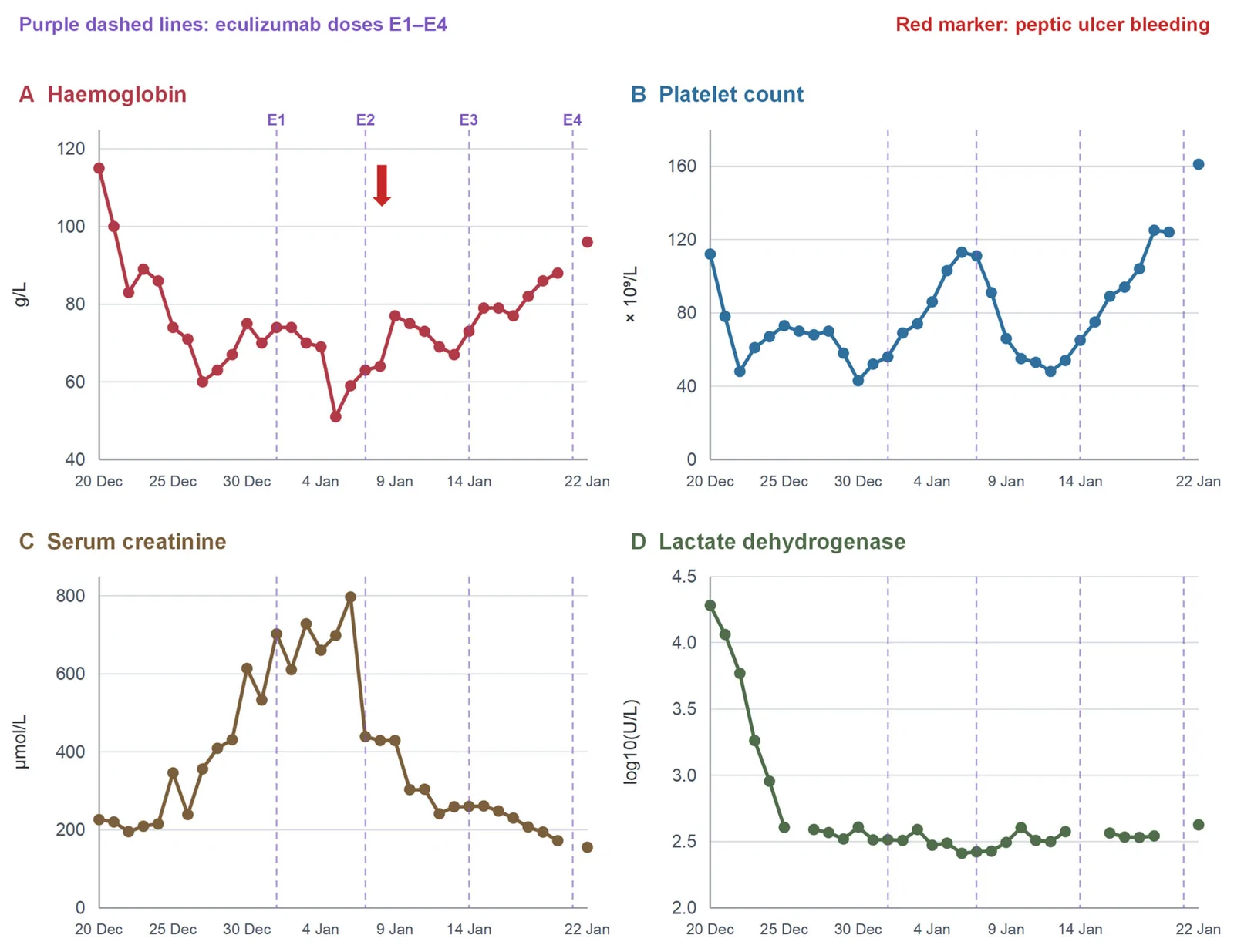
Longitudinal haematological and renal trajectories from 20 December 2025 to 22 January 2026. Panels show (**A**) haemoglobin, (**B**) platelet count, (**C**) serum creatinine, and (**D**) lactate dehydrogenase (LDH), expressed as log10-transformed values of LDH activity in U/L. Purple dashed vertical lines indicate eculizumab administrations (E1–E4); the red arrow in panel A marks endoscopically confirmed peptic ulcer bleeding on 7 January 2026.

## Data Availability

De-identified data supporting this case report are available from the corresponding author on reasonable request, subject to patient confidentiality and institutional requirements.

## Supplementary Materials

The following supporting information can be downloaded at: https://www.mdpi.com/article/10.3390/jcm15197417/s1, Supplementary Figure S1: Emergency coronary angiography performed on 20 December 2025; Supplementary Figure S2: Serial transthoracic echocardiographic findings during acute cardiac dysfunction and early recovery; Supplementary Figure S3: Serial chest computed tomography findings.

## Abbreviations

aHUS, atypical haemolytic uraemic syndrome; CRRT, continuous renal replacement therapy; IABP, intra-aortic balloon pump; LDH, lactate dehydrogenase; LVEF, left ventricular ejection fraction; mNGS, metagenomic next-generation sequencing; TMA, thrombotic microangiopathy; TTP, thrombotic thrombocytopenic purpura; VA-ECMO, venoarterial extracorporeal membrane oxygenation; VT/VF, ventricular tachycardia/ventricular fibrillation.
