# Supplementary material for: Legionella Septic Cardiomyopathy Requiring Venoarterial Extracorporeal Membrane Oxygenation Followed by Presumed Complement-Mediated Thrombotic Microangiopathy: A Case Report
Source: J Clin Med. 2026 Sep 24;15(19):7417. doi: 10.3390/jcm15197417 (PMC13666282; doi:10.3390/jcm15197417)

Supplementary Materials

Legionella Septic Cardiomyopathy Requiring VA-ECMO Followed by Presumed Complement-Mediated Thrombotic Microangiopathy: A Case Report

Supplementary Figure S1. Emergency coronary angiography performed on 20 December 2025. (A) Representative angiographic views of the right coronary artery (RCA). (B,C,D) Representative views of the left coronary artery (LCA). Previously implanted coronary stents remained patent, with no acute coronary occlusion identified. RCA, right coronary artery; LCA, left coronary artery.

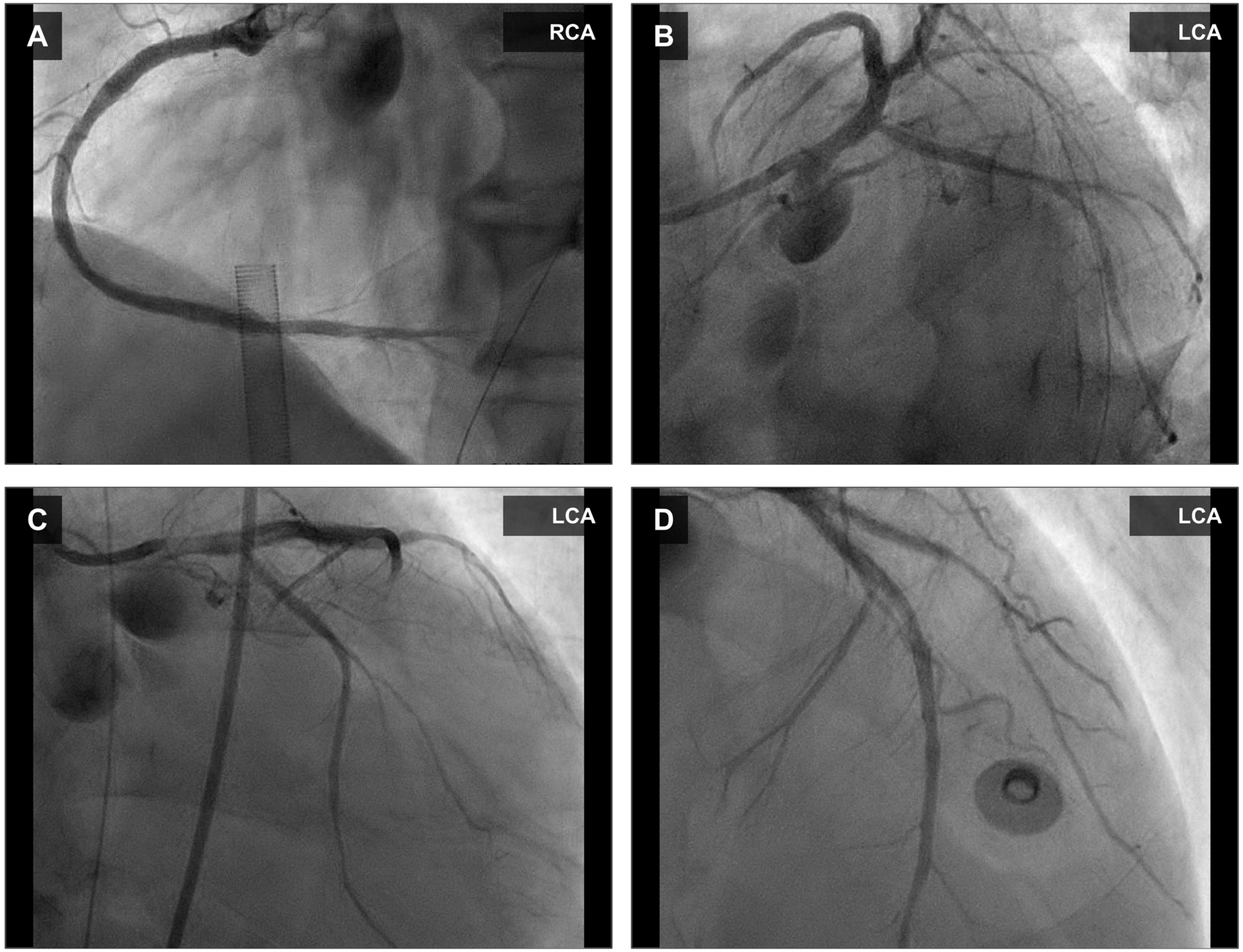

Supplementary Figure S2. Serial transthoracic echocardiographic findings during acute cardiac dysfunction and early recovery. (A–C) Parasternal long-axis, parasternal short-axis, and apical four-chamber views obtained on 20 December 2025 during the acute shock phase, when echocardiography demonstrated diffuse biventricular hypokinesia with a left ventricular ejection fraction (LVEF) of 18%. (D–F) Corresponding views obtained on 23 December 2025 during early haemodynamic recovery. LVEF subsequently recovered to 59% at three-month follow-up on 27 March 2026. LVEF, left ventricular ejection fraction.

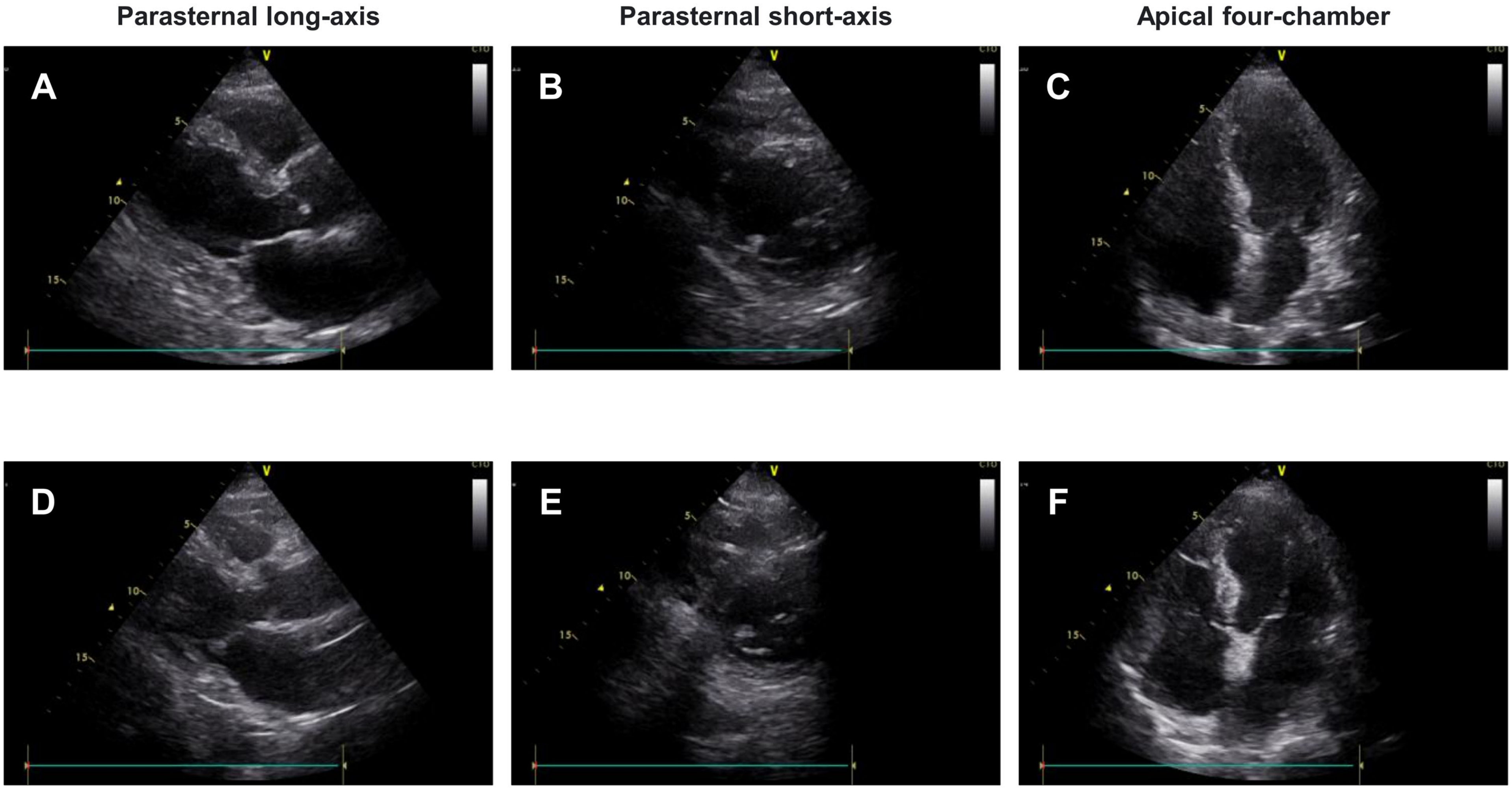

Supplementary Figure S3. Serial chest computed tomography findings. (A) Axial chest CT image obtained on 20 December 2025 showing left-predominant pulmonary consolidation. (B) Follow-up chest CT image obtained on 13 January 2026 showing substantial interval improvement of the pulmonary opacities. CT, computed tomography.

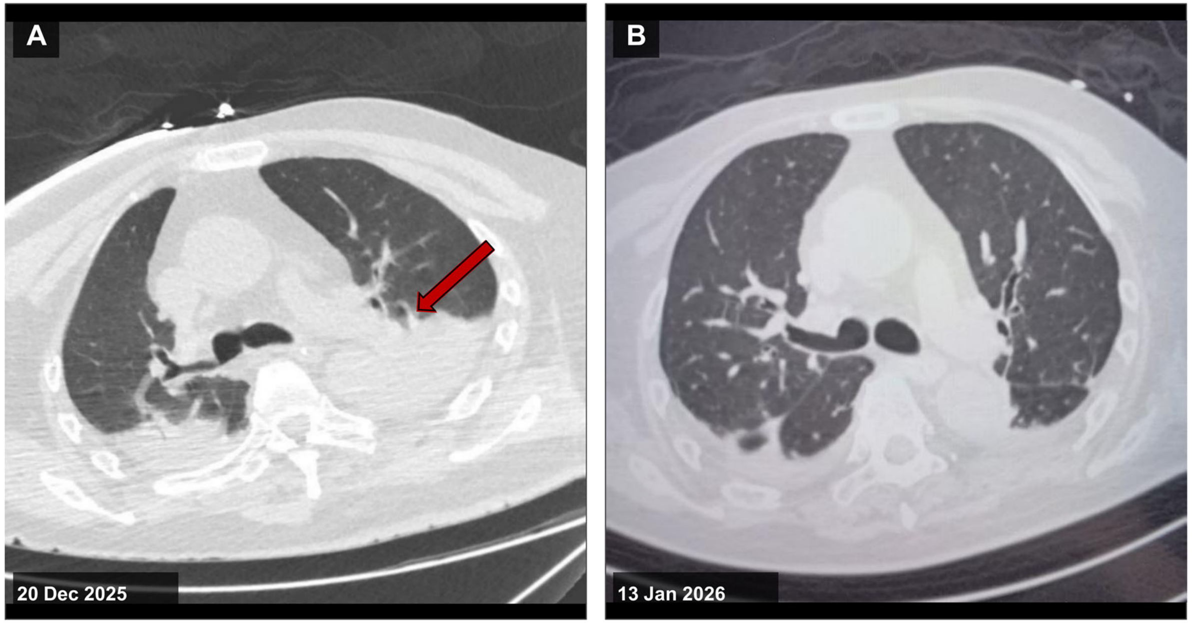

Supplement: Supplementary file 1 [file jcm-15-07417-s001.zip › JCM_supplementary.pdf]
